# Nucleotide Sequence Diversity and Linkage Disequilibrium of Four Nuclear Loci in Foxtail Millet (*Setaria italica*)

**DOI:** 10.1371/journal.pone.0137088

**Published:** 2015-09-01

**Authors:** Shui-lian He, Yang Yang, Peter L. Morrell, Ting-shuang Yi

**Affiliations:** 1 China Southwestern Germplasm Bank of Wild Species, the Key Laboratory of Biodiversity and Biogeography, Kunming Institute of Botany, Chinese Academy of Sciences, Kunming, Yunnan, China; 2 Landscape and Horticulture College, Yunnan Agriculture University, Kunming, Yunnan, China; 3 Department of Agronomy & Plant genetics, 411 Borlaug Hall, 1991 Upper Buford Circle, University of Minnesota, Saint Paul, Minnesota, United States of America; National Institute of Plant Genome Research, INDIA

## Abstract

Foxtail millet (*Setaria italica* (L.) Beauv) is one of the earliest domesticated grains, which has been cultivated in northern China by 8,700 years before present (YBP) and across Eurasia by 4,000 YBP. Owing to a small genome and diploid nature, foxtail millet is a tractable model crop for studying functional genomics of millets and bioenergy grasses. In this study, we examined nucleotide sequence diversity, geographic structure, and levels of linkage disequilibrium at four nuclear loci (*ADH1*, *G3PDH*, *IGS1* and *TPI1*) in representative samples of 311 landrace accessions across its cultivated range. Higher levels of nucleotide sequence and haplotype diversity were observed in samples from China relative to other sampled regions. Genetic assignment analysis classified the accessions into seven clusters based on nucleotide sequence polymorphisms. Intralocus LD decayed rapidly to half the initial value within ~1.2 kb or less.

## Introduction

Since the inception of the human agropastoral transition, many crops have been domesticated and served as important food sources. In East Asia, foxtail millet, known in Chinese as ‘xiao mi’ or ‘su’ is one of the most important and earliest domesticated grain crops. The earliest archeological evidence of domesticated foxtail millet was found at the Nanzhuangtou (11.5–11.0 YBP) and Donghulin (11.0–9.5 YBP) in the North China Plain [1]. Foxtail millet played an important role in early agriculture in temperate Asia and Europe [2, 3]. Worldwide, foxtail millet is a minor and regional crop today. Nonetheless, foxtail millet is still widely cultivated in Asia, Europe, North America, Australia and North Africa as a grain for human consumption or forage [4]. Foxtail millet remains a staple food in some arid regions, particularly in northern China [5]. With highly productive cultivars being developed recently, foxtail millet is seeing a resurgence in terms of areas and levels of cultivation in China and also other regions in the world [6].

There is also renewed interest in foxtail millet as a model system for bioenergy research [7–9]. Just as *Brachypodium distachium* has been sequenced as a means of providing more ready access to the large genomes of Triticeae species such as barley (*Hordeum vulgare* L.), rye (*Secale cereale* L.), and wheat (*Triticum aestivum* L.) [10], foxtail millet is a tractable genetic model for large genome, biofuel crops in the Paniceae including switchgrass (*Panicum virgatum* L.), pearl millet (*Pennisetum glaucum* (L.) R.Br.), and napiergrass (*Pennisetum purpureum* Schumach.) [11]. With an inbreeding mating system and as a true diploid with a genome size of 407.5 MB (http://www.phytozome.net/Setariaitalica), short generation time makes foxtail millet a readily tractable system to rapidly explore the genetic basis of resistance to abiotic stress, agronomic traits, and phenotypic variation in Paniceae grasses that have applications as bioenergy crops [10–12]. A major breakthrough in *Setaria* genome research is the release of the first assembled reference genome of cultivated and wild foxtail millet [13, 14], which significantly promoted improvement programs both as a food source and a model system for exploring bioenergy grasses. The reference genomes will also assist in deciphering the sequence variations across species and associated useful traits [8]. Several studies have been carried out on genome-wide analyses to develop polymorphic markers for large-scale genotyping applications [15]; identify SNPs and insertion/deletion polymorphisms through re-sequencing [16]; identify phenotype-genotype associations [17].

Both association studies [18] and genomic scans [19] for targets of selection have become important tools for the identification of the genes responsible for complex trait variation [20, 21]. Nucleotide diversity, geographic structure and level of linkage disequilibrium (LD) impact the effectiveness of both association studies [18] and scans for selection [19]. The decay of LD will determine the number and density of markers and appropriate experimental design for association analysis [22, 23] and the genomic extent of a selective sweep [19]. Owing to higher levels of homozygosity and less effective recombination, inbreeding species are expected to have higher levels of LD [24]. However, a few of previous studies obtained inconsistent results about the extent of LD in foxtail millet [25]. We used four nuclear loci to investigate the magnitude and patterns of LD for foxtail millet.

Levels of nucleotide sequence diversity and geographic structure are important in association studies [18, 23], subpopulations can result in spurious association due to confounding of unlinked markers with phenotypic variation [26]. Genetic diversity of foxtail millet has been addressed using morphology [27], isozymes [28, 29], RAPDs [30, 31], AFLP [32, 33], SSRs [34, 35], eSSRs [36], ILPs [15], RFLP [37], ribosomal DNA [38–40] and transposable elements based markers [7]. Applying multiple single copy nuclear genes and extensive sampling, we can examine genetic diversity of foxtail millet across its range of cultivation.

Applying a collection of 311 foxtail millet accessions across its historical range of cultivation, we used resequencing data from four loci to: (1) document the nucleotide diversity within and among geographic regions; (2) examine the genetic structure; (3) investigate the extent of intralocus linkage disequilibrium (LD). Based on this information, the application of association studies in foxtail millet was addressed.

## Material and Methods

### Samples

A total of 311 foxtail millet landrace accessions were sampled. China, Central Asia, and European accessions constitute a large portion of the total sample because these regions have been the major regions of cultivation and/or putative domesticated centers (S1 Table). Seeds for each accession were drawn from the collections of the United States Department of Agriculture (USDA), the National Institute of Agrobiological Sciences (NIAS), and the Chinese Academy of Agricultural Science (CAAS).

Genomic DNA was extracted from young leaf tissue from a single individual of each accession using the modified CTAB method [41]. We examined four nuclear loci: Trisephosphate isomerase 1 (*TPI1*), glyceraldehyde 3-phosphate dehydrogenase (*G3PDH*), Alcohol dehydrogenase 1 (*ADH1*), the intergenic spacer 1 (*IGS1*) region of ribosomal DNA. Because the *IGS1* region is a portion of nuclear ribosomal DNA that occurs as tandem array, which is subjected to concerted evolution [42], we compared diversity at *IGS1* among geographic regions but did not include it in estimates of average diversity. The PCR and sequencing primers for *ADH1*, *G3PDH*, *IGS1* and *TPI1* were designed from *Zea mays*. Primer information is detailed in Table 1. These primers were located in conserved portions of the genes when comparing the sequences between *Oryza sativa* and *Zea mays*, thus these primers have potential utility across the Poaceae.

**Table 1:** Primers’ information for four loci.

gene	encoding protein	maize gene ID	foxtail millet gene ID	primer name	sequence (5'->3')	type of primer
*IGS1*	The ribosomal intergenic spacer1	-	-	IGS1-1	CATTGTAAGTGGCAGAGTGG(Tm = 57.80)	PCR & sequencing
				IGS1-3	TGACTACTGGCAGGATCAAC(Tm = 57.80)	PCR & sequencing
*ADH1*	Alcohol Dehydrogenase 1	542363	101767284	Adh1-1	ATYTgCTCAggATCAACACT(Tm = 54.73)	PCR & sequencing
				Adh1-4	gTgATgAACTTCTCCACCTC(Tm = 57.80)	PCR & sequencing
*G3PDH*	Glyceraldehyde 3 phosphate dehydrogenase	103642790	101785151	G3pdh-1	GTTTTGTGGTGGGTTCAG(Tm = 55.02)	PCR & sequencing
				G3pdh-4	CTTCCACCTCTCCAGTCC(Tm = 59.58)	PCR & sequencing
				G3pdh-3	GAAGAGTCCAATAACTCTGCTT(Tm = 56.35)	sequencing
*TPI1*	Triosephosphate isomerase 1	103645706	101778953	TPI1-F	GCAACTGGAAATGCGTAA(Tm = 52.74)	PCR & sequencing
				TPI1-R	AGCACCTCCCTTCTTCAC(Tm = 57.3)	PCR & sequencing
				TPI1-M	TATGGATCTCCAGAAGTTGG(Tm = 55.75)	sequencing

### PCR and DNA sequencing

Polymerase chain reaction (PCR) was performed in a total volume of 25 μl containing 50 ng of template DNA, 10 μM of each primer, 2.5 μL 10 × PCR buffer (Mg^2+^), 2.5 mM of dNTP, and 1 unit of Taq DNA polymerase. PCR reactions (except *G3PDH*) used the following cycling conditions: 94°C for 3 min; 35 cycles (94°C for 80 sec, 54°C for 90 sec, 72°C for 90 sec); a final extension of 10 min at 72°C, the annealing temperature of *G3PDH* is 56°C. Amplified products were purified with EasyPure PCR Purification Kit (TransGen, Beijing, China), and were directly sequenced on an ABI (Applied Biosystems, Foster City, California, USA) 3730. The majority of PCR amplicons were directly sequenced. Amplicons from accessions that were determined to be heterozygous within a locus were cloned using pEASY kit (TransGen, Beijing, China). Not all samples were sequenced successfully, thus the sample size of final data matrix for *ADH1*, *G3PDH*, *IGS1*, *TPI1* are 296, 285, 293, and 289, respectively.

Sequence for each accession was assembled in Sequencher 4.1.4 (Gene Codes Corp, Ann Arbor, MI, USA), and edited sequences were aligned using Clustal X [43], and were further adjusted manually. The accuracy of haplotype data was assessed using Error Detection Using Triplets (EDUT) [44]. We carried out resequencing when private SNP occurred.

### Sequences Analysis

#### Sequence Diversity Estimation and Tests of Neutrality

Descriptive statistics for nucleotide sequence diversity and tests of neutrality were estimated using tools from the libsequence C++ software library [45]. Insert/deletion polymorphism was treated as missing data. We reported two estimates of *θ* = 4*N*_e_ *μ*, based on the number of segregating sites (*θ*_*w*_) [46] and the number of pairwise differences among haplotypes (*θ*_*π*_) [47]. We also reported Tajima’s *T* [48], a test of departures from neutrality under a standard coalescent model. The significance of Tajima’s *T* was tested by 10,000 replicate coalescent simulations.

#### Geographic structure

The sample was partitioned into nine geographic regions: China (CH), Central Asia (CA), South Asia (SA), Near East (NE), Korea and Japan (KJ), Southeast Asia (SEA), Europe (ER), North America (NA), and Africa (AF). We tested the extent of geographic structure in and among these partitions of the sample using *K*_ST_* and *S*_nn_ methods of Hudson [49, 50] applied programs implemented in libsequence C++ software library [45]. *S*_nn_ (nearest-neighbor statistic) is a powerful statistic for detecting genetic differentiation using sequence-based analysis over a wide range of sample size and levels of variation [49]. If two populations are highly differentiated, *S*_nn_ is expected to be near one. *K*_ST_* measures genetic differentiation using resequencing data and can be more powerful for detecting population structure than SNP frequency-based estimates of *F*_ST_, especially in the case of high haplotype diversity [49, 51].

Genetic differentiation was investigated using genetic assignment in STRUCTURE 2.3.4 [52, 53]. Burn-in time and replication number were set to 50,000 and 50,000 for each run, respectively. The number of populations (*K*) in the model was systematically varied from 1 to 10, with the median likelihood of each *K* value estimated from the 20 runs. We used the △*K* method [54] representing the highest median likelihood values using the online service Structure Harvester [55].

### Linkage Disequilibrium Analysis

Multiple methods were applied to estimate levels of LD in the data: a parametric estimate of the recombination rate: *ρ* = 4*N*_e_ *r*, where *N*_e_ is the effective population size and *r* is the recombination rate per generation [56, 57]; a composite likelihood estimator, based on pairwise LD between sites [58]; and intralocus LD estimating using Wall’s *B* [59], a summary statistics with values approaching 1 indicating extensive congruence among adjacent segregating sites. The minimum number of recombination events (*R*_*m*_) [60] were also reported.

LD between pairs of polymorphic sites was calculated based on squared correlation in allele frequency, *r* ^2^ [61], between each pair of SNPs with a frequency filter of ≥ 20%. The decay of LD with physical distance was estimated using nonlinear regression of LD between polymorphic sites versus distance in base pairs between sites [62]. The statistical package R (http://www r-project.org) was used to plot LD versus distance.

## Results

### Nucleotide Sequence Polymorphism and Diversity

**Fig 1:**
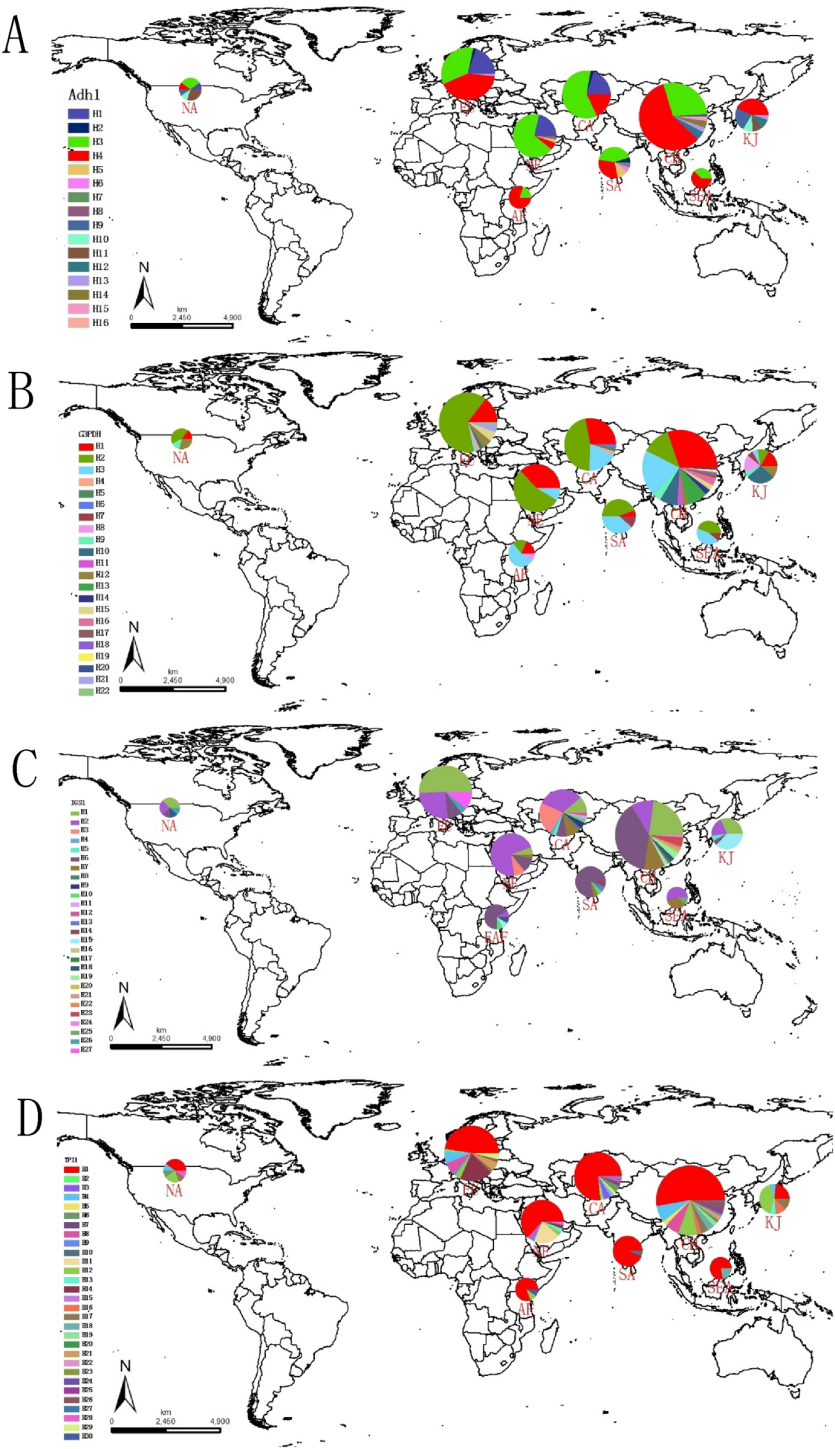
Geographic distribution of haplotypes of foxtail millet. The distribution of haplotypes for the (A) *ADH1*, (B) *G3PDH*, (C) *IGS1*, (D) *TPI1* locus are shown. Each distributed regions are indicated by circles. Different colors delineate specific haplotypes. The size of the circles in the figure corresponds to numbers of accessions in a given region.

The aligned sequence of *ADH1*, *G3PDH*, *IGS1*, and *TPI1* are 875, 1390, 970, and 870 bp, respectively, and the combined total length of aligned sequence for four loci is 4105 bp (Table 2). Most foxtail millet individuals were found to be homozygous at the four nuclear loci used in this study, and most amplicons could be sequenced directly except two individuals heterozygous for *TPI1* and three for *ADH1*. These results are consisted with previous study that foxtail millet has a high selfing mating system, but also have a crossing rate of 0.002 to 0.6% [25]. For the amplicons could not be sequence directly, we applied cloning method to infer individual haplotypes. There are 16, 22, 27 and 30 haplotypes detected from *ADH1*, *G3PDH*, *IGS1*, and *TPI1*, respectively (Table 2, S2 Table, Fig 1).

**Table 2:** Estimate of nucleotide sequence diversity, Tajima’s *T* (commonly reported as Tajima’s *D* test), Wall’s *B*, and *R*_m_ for 311 accessions of foxtail millet.

Gene	Length, bp	Region	*n*	*s*	*h*	haplotype diversity	*θ*_*W*_ x 10^3^	H_01_ x10^3^	*θ*_*π*_ x 10^3^	*θ*_π (replacement)_ x 10^3^	*θ*_π Silent Polymorphism_x 10^3^	T	Wall’s *B*	R_m_
*ADH1*	875	All sequences	296	22	16	0.69	4.17	NA	3.83	5.49	0.02	-0.21	0.09	2
		CA	45	7	4	0.58	1.83	1.34	3.60	0.00	0.00	2.62	0.33	1
		ER	59	15	7	0.72	3.70	1.06	3.62	0.00	0.00	-0.06	0.21	1
		KJ	21	16	6	0.77	5.09	NA	4.30	0.00	0.01	-0.57	0.27	0
		NA	7	8	4	0.81	3.73	NA	4.46	0.00	0.01	1.03	0.43	0
		NE	37	7	5	0.51	1.92	1.66	3.35	0.00	0.00	2.11	0.33	1
		SA	19	8	6	0.74	2.62	NA	3.44	0.00	0.00	1.07	0.29	1
		SEA	8	5	2	0.54	2.20	NA	3.06	0.00	0.00	1.76	1.00	0
		CH	90	15	9	0.56	3.51	0	3.17	0.13	0.00	-0.27	0.14	0
		AF	10	6	2	0.60	2.42	NA	2.90	NA	NA	0.80	0.60	0
*G3PDH*	1390	All sequences	285	34	22	0.78	4.09	NA	4.20	1.18	4.80	0.07	0.06	4
		CA	43	18	6	0.69	3.02	1.84	4.02	1.00	4.93	1.07	0.12	2
		CH	87	33	15	0.84	4.90	0.99	6.01	1.45	7.03	0.71	0.13	3
		ER	56	32	10	0.59	5.06	3.92	2.14	0.49	2.63	-1.90	0.16	2
		KJ	19	29	9	0.89	6.02	NA	6.09	1.46	7.50	0.05	0.18	0
		NA	7	5	4	0.81	1.48	NA	1.38	0.00	1.81	-0.33	0.25	0
		NE	35	16	3	0.58	2.82	0	2.59	0.48	3.23	-0.27	0.60	0
		SA	18	17	5	0.71	3.58	NA	5.38	1.57	6.54	1.92	0.38	0
		SEA	9	15	3	0.67	3.98	NA	5.76	1.66	7.07	2.17	0.86	0
		AF	11	16	4	0.69	3.96	NA	5.85	5.09	6.13	2.13	0.60	0
*TPI1*	870	All sequences	289	21	30	0.65	3.87	NA	2.86	4.01	2.75	-0.68	0.00	3
		CA	43	11	9	0.41	2.91	4.20	1.02	0.00	1.09	-1.94	0.20	1
		CH	91	17	17	0.71	3.84	9.17	3.20	3.60	3.08	-0.47	0.00	2
		ER	56	13	11	0.73	3.24	3.68	2.34	2.90	2.29	-0.82	0.00	1
		KJ	17	8	6	0.79	2.70	NA	3.63	5.18	3.24	1.21	0.14	1
		NA	9	9	5	0.81	3.78	NA	4.57	9.73	4.20	0.96	0.38	0
		NE	35	10	8	0.58	2.77	5.132	3.02	2.83	3.02	0.27	0.00	1
		SA	20	7	3	0.28	2.25	NA	1.19	1.75	1.15	-1.54	0.17	0
		SEA	9	7	2	0.42	2.94	NA	2.91	0.00	3.11	-0.03	0.83	0
		AF	11	7	4	0.49	2.72	NA	2.57	5.73	2.35	-0.23	0.67	0
*IGS1*	970	All sequences	293	76	27	0.82	18.72	NA	10.05	NA	NA	-1.38	0.111	5
		CA	44	44	12	0.84	14.99	0	12.81	NA	NA	-0.51	0.26	0
		CH	92	56	14	0.78	16.34	3.18	11.53	NA	NA	-0.95	0.13	3
		ER	56	14	6	0.68	4.26	6.38	4.73	NA	NA	0.33	0.23	3
		KJ	19	12	5	0.75	4.76	NA	5.82	NA	NA	0.81	0.27	0
		NA	8	12	5	0.86	6.45	NA	5.77	NA	NA	-0.53	0.18	0
		NE	35	32	5	0.48	11.26	0	6.32	NA	NA	-1.55	0.55	0
		SA	19	34	5	0.46	14.51	NA	6.80	NA	NA	-2.13	0.32	0
		SEA	9	31	3	0.64	17.10	NA	20.42	NA	NA	0.98	0.43	0
		AF	11	12	4	0.49	5.69	NA	3.74	NA	NA	-1.49	0.27	0

Notes: 1. CA: Central Asia; NE: Near East; KJ: Korea/Japan; SA: South Asia; CH: China; NA: North America; EU: Europe; SEA: Southeast Asia; AF: Africa. 2. *n* = number of accessions; *s* = number of SNPs; *h* = number of haplotypes; *θ*_*π*_ = pairwise nucleotide diversity; *θ*_*w*_ = nucleotide diversity; *T* = Tajima’s *T*.

Nucleotide diversity at each locus is shown in Table 2. The clear sequence alignment profile is shown in S1 Fig. A total of 152 SNPs were detected across the four loci. For the three single copy genes, diversity per locus ranged from *θ*_*w*_ = 3.89–4.09 × 10^−3^ with an average *θ*_*w*_ = 3.98 × 10^−3^. The nucleotide diversity of locus *IGS1* is 18.72 × 10^−3^, which is much higher than three single copy genes. Tajima’s *T* value for *ADH1*, *G3PDH*, *IGS1* and *TPI1* are -0.14, 0.07, -0.75 and -1.38 (*P* > 0.1), respectively. None of the Tajima’s *T* values show significant deviation from expectations for a panmictic population evolving under neutrality. Nucleotide diversity estimated by the number of pairwise differences among haplotypes (*θ*_*π*_) ranges from 2.86–10.05 × 10^−3^ for the four loci, with on average of 6.98 × 10^−3^ (Table 2).

Among the four most extensively sampled regions including Central Asia, the Near East, China, and Europe, accessions from China have the highest average nucleotide diversity at four loci measured by *θ*_*w*_ except *G3PDH* and by *θ*_*π*_ except *ADH1* (Table 2). Excluding these regions, the Korea-Japan region has especially high nucleotide diversity, and has higher nucleotide diversity than that of China at four loci except *G3PDH*.

### Geographic Structure

Results of the *S*_nn_ test for geographic structure and estimates of *K*_ST_* at each locus are shown in Table 3. *S*_nn_ indicates significant geographic structure (*P* < 0.001) for all four loci. *K*_ST_*statistics in locus of *IGS1* (*P* < 0.05) also demonstrates significant geography structure (Table 3).

**Table 3:** Significance of *K*_ST_* and *S*_nn_ tests for geographic structure.

Gene	sample	K_ST_*	P-value	S_nn_	P-value
*ADH1*	All samples	-0.0014156	0.4737	0.245388	0
*G3PDH*		0.12011	0.0715	0.268825	0
*IGS1*		0.13964	0.0175	0.325848	0
*TPI1*		0.0709283	0.1077	0.264668	0

**Fig 2:**
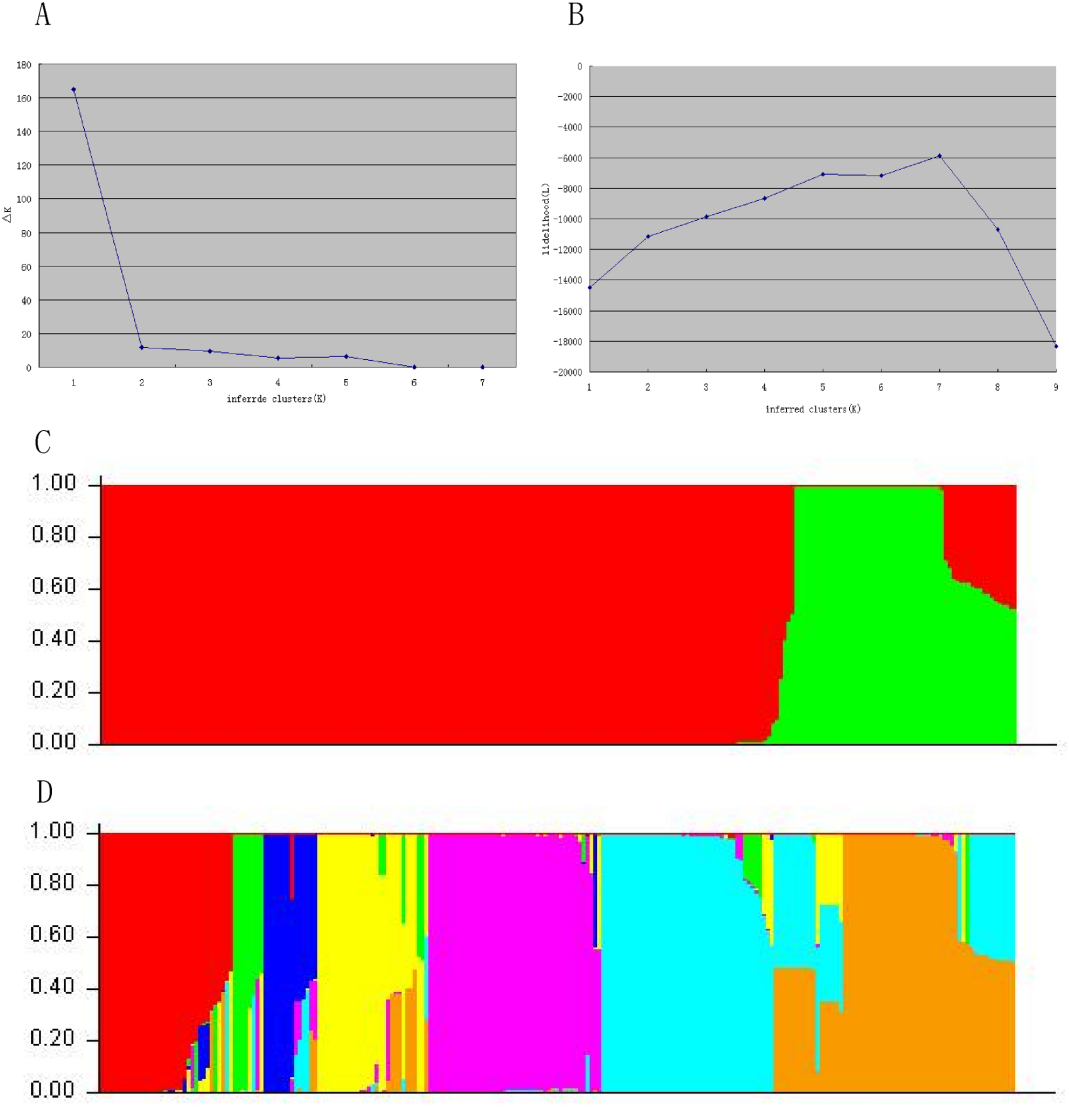
Structure analysis. The median *△K* for 10 runs for each *K* estimate are shown. A. median likelihood values. B. Population subdivision and the frequency distribution of foxtail millet population in each inferred clusters. Results from both the *K* = 2 (C) and *K* = 7 (D) models are shown. Each accession is shown by a thin vertical line that is partitioned into two or seven colored segments. The accessions in which membership probability is < 50% are classified into a “mixed” group.

Genetic assignment based on the four loci showed moderate genetic structure. Evanno’s [54] ad hoc estimator of the actual number of clusters was used, and the results showed *△K* indicate modes at *△K* = 2 & 7 model best fit the data, suggesting that the sample can be divided into 2 or 7 clusters. We examined the geographic distribution using both the *K* = 2 and *K* = 7 models (Fig 2).

### Estimation of Recombination and Linkage Disequilibrium

Estimates of the recombination parameter *ρ = 4N*_*e*_ *r* for each population are shown in Table 2. Recombination rate estimates are commonly lower than estimates of mutation rate *θ*_*w*_ for most loci except for the *TPI1* locus.

**Fig 3:**
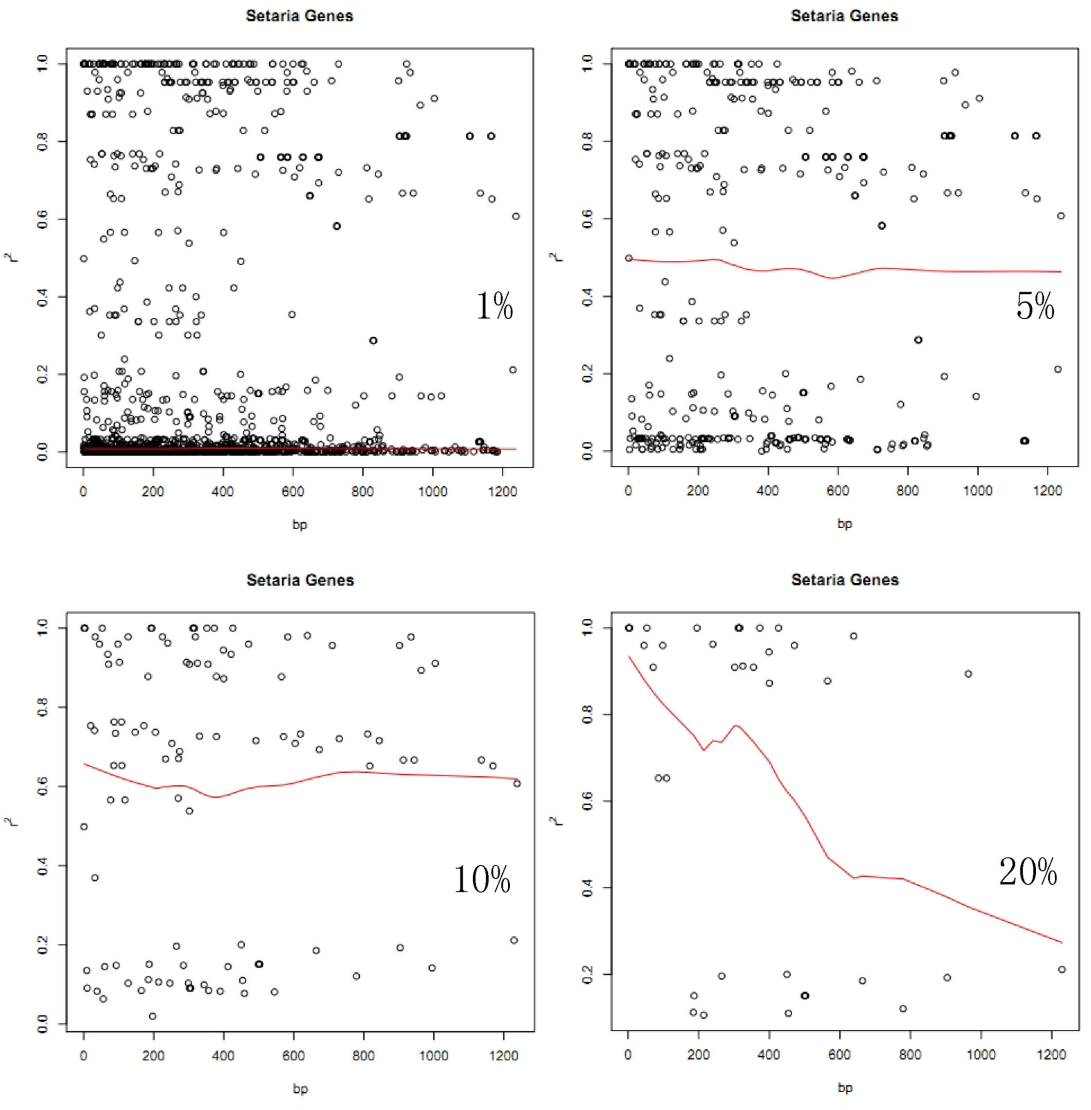
Plots of squared allele frequency correlations (*r* ^2^) by physical distance between sites in foxtail millet. An SNP frequency filter of 1%, 5%, 10%, 20% was used. The red line in each plot depicts the lowess smoothed line that summarized the observed data in LD.

Plots of squared allele frequency correlations (*r* ^2^) by physical distance between sites in foxtail millet are shown in Fig 3. SNP frequency filters of 1%, 5%, 10%, 20% were used. The red line in each plot depicts the lowess smoothed line that summarized the observed LD data. Intralocus LD is shown in the plot of *r* ^2^ against distance in base pairs between SNPs (Fig 3). Nonlinear regression shows clear and rapid decline of LD with distance in Fig 3, and the LD decays rapidly to half the initial value within ca. 1.2 kb. Wall’s *B* value (0–0.21) also suggests similar pattern of intralocus LD.

## Discussion

### Genetic Diversity and Geographic Structure in Foxtail Millet

Foxtail millet has relatively low diversity for the three single copy genes (*ADH1*, *G3PDH*, *TPI1*) with average diversity *θ*_*w*_ = 4.04 × 10^−3^, *θ*_*π*_ = 3.63 × 10^−3^. The level of genetic diversity is lower than wild green foxtail (*Setaria viridis* (L.) P. Beauv.), where *θ*_*w*_ = 5.9 × 10^−3^ [63]. However, despite the reduction in diversity relative to the wild progenitor species, foxtail millet still harbores a slightly higher level of diversity compared with other domesticated crop, such as rice (*Oryza sativa*) (*θ*_*π*_ = 3.04 × 10^−3^) [64]. There were also some previous studies for genetic diversity of foxtail millet through variable methods. For example, Wang et al. surveyed DNA sequence for nine loci across 50 accessions of cultivated foxtail millet and found lower value of Watterson’s estimator (2.70 × 10^−3^) [12], Liu et al. screened 128 accessions with 79 SSR markers and found the mean genetic diversity was 0.75 and the mean polymorphism information content (PIC) was 0.72 [65]. Then they identified genetic diversity of 111 accessions applied 23 mitochondrial DNA loci and revealed the mean genetic diversity was 0.29 and mean PIC was 0.23 [66]. Many genetic markers were developed including the Intron-Length Polymorphic Markers [15], SSR markers [34, 35], transposable elements-based markers (TEs) [7], which could be applied in genetic diversity study of foxtail millet in the future.

Cytological and genetic studies indicated that the wild ancestor of this crop is *S*. *viridis* [32, 67–69]. However, the domesticated times and domesticated center of foxtail millet is still an issue of intense debate, with China, Central Asia, Europe and Near East were considered as the most possible domestication centers by different studies [39, 70–72]. These four centers are also major cultivated regions, and a heavy sampling strategy was applied. China has the highest nucleotide diversity (*θ*_*π*_) for loci *G3PDH* and *TPI1*, Europe for locus *ADH1*, and Central Asia for locus *IGS1* showed. Europe has the highest haplotype diversity for loci *ADH1* and *IGS1*, China for locus *G3PDH*, and Central Asia for locus IGS1. All the four regions except Near East contain high nucleotide diversity, haplotype diversity and private SNPs for certain locus, which should be major regions for germplasm conservation. Korea and Japan (KJ) have rarely been included in previous studies, high diversity (haplotype diversity = 0.8 × 10^−3^; *θ*_*w*_ = 4.64 × 10^−3^; *θ*_*π*_ = 6.65 × 10^−3^) was revealed in this study. The *S*_nn_ test indicates significant geographic structure for four loci studied (*P* = 0). The *ADH1* locus also show significant population structure with *K*_ST_* (*P* < 0.05). Given that the number of loci sampled is limited, the geographic structure of foxtail millet populations will require further exploration, but current data suggest a high degree of differentiation among geographic regions.

### Linkage Disequilibrium in Foxtail Millet

Generally speaking, LD decays more rapidly in outcrossing than selfing species [73] because recombination is less effective in selfing species, where individuals are more likely to be homozygous. However, mating system alone has not proven especially predictive of the extent of linkage disequilibrium in self-fertilizing species, with relatively high levels of LD observed in *Arabidopsis*. *thaliana* (within 10 kb) [74] and *Boechera stricta* (within ~ 10 kb or less) [18]; more rapid decay of LD in wild and cultivated rice (*O*. *sativa*) (< 1 kb) [75], and very limited LD in wild barley, which is 98% selfing and has levels of LD are similar to those observed at in cultivated maize (< 1 kb) [76–78]. Foxtail millet has a very low outcrossing rate, from 0.002 to 0.6%. However, intralocus LD at four loci show a significant negative correlation of LD with physical distance and decays to less than half the initial value within 1 kb. The decay of LD is most evident when the frequency filter applied to the data is highest (Fig 3), a result consistent with the geographic structure discussed above.

Our results show that low levels of LD in inbreeding organisms are not exceptional and could be explained by the following hypotheses. First, low LD may be resulted from a species-wide scale of sampling, which incorporates the entire history of polymorphism and recombination within a species over thousands of generations [74, 75, 77]. With a whole cultivation region sampling, our data is consistent with this sample-scale explanation. Second, low LD may be caused when selection favoring recombinant genotypes with new combinations of parental traits [79]. This explanation is plausible for crops experienced strong selection by human beings, such as cereals, a mechanism to eliminate deleterious mutations may be existent [80, 81]. Experiments are needed on natural populations to verify fitness advantages for recombinant genotypes. These analyses may explain the low levels of LD in foxtail millet, however the actually reasons for low level of LD detected need further studies.

There are also some previous studies about linkage disequilibrium of foxtail millet. Wang et al. reported similar level of LD as ours (extends to 1 kb) applied 9 gene loci [12]. However, some previous studies applied genomic data obtained much higher LD decay rate. Applying 916 diverse foxtail millet varieties, Jia et al. used 0.8 million common SNPs to reveal the genome-wide LD decay rate was ~100 kb on average [17]. Vetriventhan et al. genotyped 155 accessions using 72 SSR markers and showed that LD decay < 40 cM of genetic distance [82]. Wang et al. reported the LD decay of less than 20 cM of genetic distance using SSR markers with 250 foxtail millet landraces [83]. Different molecular data and sampling strategy obtained inconsistent results. More analyses based on genomic data are needed to estimate sequence diversity and LD of foxtail millet.

## Supporting Information

S1 Fig Sequence alignment profile. (TIF)

S1 Table Sampling and haplotype information for domesticated foxtail millet. GG = geographic group.
(PDF)

S2 Table Accession numbers of haplotypes. (PDF)

## References

[1] Yang X, Wan Z, Perry L, Lu H, Wang Q, Zhao C, et al Early millet use in northern China. Proceedings of the National Academy of Sciences. 2012;10:3726–30. 10.1073/pnas.1115430109 PMC3309722 22355109

[2] Nasu H, Momohara A, Yasuda Y, He JJ. The occurrence and identification of Setaria italica (L.) P. Beauv. (foxtail millet) grains from the Chengtoushan site (ca. 5800 cal BP) in central China, with reference to the domestication centre in Asia. Veg Hist Archaeobot. 2007;16(6):481–94. 10.1007/s00334-006-0068-4. ISI:000248834800005.

[3] Sakamoto S. Origin and dispersal of common millet and foxtail millet. Jarq-Jpn Agr Res Q. 1987;21(2):84–9. ISI:A1987L090900002.

[4] Austin DF. Foxtail millets (Setaria: Poaceae)—abandoned food in two hemispheres. Economic Botany. 2006;60(2):143–58.

[5] Li Y, Wu SZ, Cao YS, Zhang XZ. A phenotypic diversity analysis of foxtail millet (Setaria italica (L.) P. Beauv.) landraces of Chinese origin. Genetic Resources and Crop Evolution. 1996;43(4):377–84. ISI:A1996VF75400011.

[6] Sun X. "An introduction of "Zhang Za Gu" series hybrid millet varieties. Seed World. 2009;4:47–8. 90354X.

[7] Yadav CB, Bonthala VS, Muthamilarasan M, Pandey G, Khan Y, Prasad M. Genome-wide development of transposable elements-based markers in foxtail millet and construction of an integrated database. DNA Research. 2014:dsu039. 10.1093/dnares/dsu039 PMC4379977 25428892

[8] Lata C, Gupta S, Prasad M. Foxtail millet: a model crop for genetic and genomic studies in bioenergy grasses. Critical reviews in biotechnology. 2013;33(3):328–43. 10.3109/07388551.2012.716809 22985089

[9] Muthamilarasan M, Prasad M. Advances in *Setaria* genomics for genetic improvement of cereals and bioenergy grasses. Theoretical and Applied Genetics. 2015;128(1):1–14. 10.1007/s00122-014-2399-3 25239219

[10] Bevan MW, Garvin DF, Vogel JP. *Brachypodium distachyon* genomics for sustainable food and fuel production. Curr Opin Biotech. 2010;21(2):211–7. 10.1016/j.copbio.2010.03.006. ISI:000278303100014. 20362425

[11] Doust AN, Kellogg EA, Devos KM, Bennetzen JL. Foxtail millet: A sequence-driven grass model system. Plant Physiol. 2009;149(1):137–41. 10.1104/pp.108.129627. ISI:000262261500019. 19126705 PMC2613750

[12] Wang CF, Chen JF, Zhi H, Yang L, Li W, Wang YF, et al Population genetics of foxtail millet and its wild ancestor. Bmc Genet. 2010;11:90 Artn 90 10.1186/1471-2156-11-90. ISI:000283495400001. 20937104 PMC2964552

[13] Zhang GY, Liu X, Quan ZW, Cheng SF, Xu X, Pan SK, et al Genome sequence of foxtail millet (Setaria italica) provides insights into grass evolution and biofuel potential. Nat Biotechnol. 2012;30(6):549–54. 10.1038/Nbt.2195. WOS:000305158600025. 22580950

[14] Bennetzen JL, Schmutz J, Wang H, Percifield R, Hawkins J, Pontaroli AC, et al Reference genome sequence of the model plant *Setaria* . Nat Biotechnol. 2012;30(6):555–61. 10.1038/Nbt.2196. WOS:000305158600026. 22580951

[15] Muthamilarasan M, Suresh BV, Pandey G, Kumari K, Parida SK, Prasad M. Development of 5123 intron-length polymorphic markers for large-scale genotyping applications in foxtail millet. DNA research. 2014;21(1):41–52. 10.1093/dnares/dst039 24086082 PMC3925393

[16] Bai H, Cao Y, Quan J, Dong L, Li Z, Zhu Y, et al Identifying the genome-wide sequence variations and developing new molecular markers for genetics research by re-sequencing a landrace cultivar of foxtail millet. PloS one. 2013;8(9):e73514 10.1371/journal.pone.0073514 24039970 PMC3769310

[17] Jia G, Huang X, Zhi H, Zhao Y, Zhao Q, Li W, et al A haplotype map of genomic variations and genome-wide association studies of agronomic traits in foxtail millet (Setaria italica). Nature genetics. 2013;45(8):957–61. 10.1038/ng.2673 23793027

[18] Song BH, Windsor AJ, Schmid KJ, Ramos-Onsins S, Schranz ME, Heidel AJ, et al Multilocus patterns of nucleotide diversity, population structure and linkage disequilibrium in *Boechera stricta*, a wild relative of *Arabidopsis* . Genetics. 2009;181(3):1021–33. 10.1534/genetics.108.095364. ISI:000270213500020. 19104077 PMC2651039

[19] Teshima KM, Coop G, Przeworski M. How reliable are empirical genomic scans for selective sweeps? Genome Res. 2006;16(6):702–12. 10.1101/Gr.5105206. ISI:000237973200002. 16687733 PMC1473181

[20] Aranzana MJ, Kim S, Zhao KY, Bakker E, Horton M, Jakob K, et al Genome-wide association mapping in *Arabidopsis* identifies previously known flowering time and pathogen resistance genes. Plos Genetics. 2005;1(5):531–9. ARTN e60 10.1371/journal.pgen.0010060. ISI:000234714900004. PMC1283159 16292355

[21] Ross-Ibarra J. Genome size and recombination in angiosperms: a second look. J Evolution Biol. 2007;20(2):800–6. 10.1111/j.1420-9101.2006.01275.x. ISI:000244244300038. 17305845

[22] Slatkin M. Linkage disequilibrium—understanding the evolutionary past and mapping the medical future. Nature Reviews Genetics. 2008;9(6):477–85. 10.1038/Nrg2361. ISI:000255953500015. 18427557 PMC5124487

[23] Flint-Garcia SA, Thornsberry JM, Buckler ES. Structure of linkage disequilibrium in plants. Annual Review of Plant Biology. 2003;54:357–74. 10.1146/annurev.arplant.54.031902.134907. ISI:000185094100014. 14502995

[24] Nordborg M, Borevitz JO, Bergelson J, Berry CC, Chory J, Hagenblad J, et al The extent of linkage disequilibrium in *Arabidopsis thaliana* . Nature Genetics. 2002;30(2):190–3. 10.1038/ng813 11780140

[25] Till-Bottraud I, Reboud X, Brabant P, Lefranc M, Rherissi B, Vedel F, et al Outcrossing and hybridization in wild and cultivated foxtail millets: consequences for the release of transgenic crops. Theoretical and Applied Genetics. 1992;83(8):940–6. ISI:000207061400002. 10.1007/BF00232954 24202917

[26] Buckler ES, Thornsberry JM, Kresovich S. Molecular diversity, structure and domestication of grasses. Genetical Research. 2001;77(3):213–8. ISI:000169948900001. 11486504 10.1017/s0016672301005158

[27] Rao KEP, Dewet JMJ, Brink DE, Mengesha MH. Infraspecific variation and systematics of cultivated Setaria italica, foxtial millet (Poaceae). Economic Botany. 1987;41(1):108–16. WOS:A1987G043800016.

[28] Jusuf M, Pernes J. Genetic variability of foxtail millet (Setaria italica P Beauv)—electrophoretic study of 5 isoenzyme systems. Theoretical and Applied Genetics. 1985;71(3):385–91. ISI:A1985AWG0400003. 10.1007/BF00251177 24247442

[29] Gao M, J, Chen JJ. Isosyme studies on the origin of cultivated foxtail millet. Acta Agronomica Sinica. 1988;14:132–6. Epub 136.

[30] Li Y, Jia JZ, Wang Y, Wu SZ. Intraspecific and interspecific variation in *Setaria* revealed by RAPD analysis. Genetic Resources and Crop Evolution. 1998;45(3):279–85. ISI:000074318600011.

[31] Schontz D, Rether B. Genetic variability in foxtail millet, Setaria italica (L.) P. Beauv.: Identification and classification of limes with RAPD markers. Pl Breed. 1999;118(2):190–2. ISI:000080523900015.

[32] d'Ennequin ML, Panaud O, Toupance B, Sarr A. Assessment of genetic relationships between *Setaria italica* and its wild relative *S*. *viridis* using AFLP markers. Theoretical and Applied Genetics. 2000;100(7):1061–6. ISI:000087586500008.

[33] Kossover O, Frenkel Z, Korol A, Nevo E. Genetic diversity and stress of Ricotia lunaria in "Evolution Canyon," Israel. Journal of Heredity. 2009;100(4):432–40. 10.1093/jhered/esp014. ISI:000266965200005. 19321630

[34] Zhang S, Tang CJ, Zhao Q, Li J, Yang LF, Qie LF, et al Development of highly polymorphic simple sequence repeat markers using genome-wide microsatellite variant analysis in Foxtail millet [Setaria italica (L.) P. Beauv.]. Bmc Genomics. 2014;15(1):78 Artn 78 10.1186/1471-2164-15-78. WOS:000332573700001. 24472631 PMC3930901

[35] Pandey G, Misra G, Kumari K, Gupta S, Parida SK, Chattopadhyay D, et al Genome-wide development and use of microsatellite markers for large-scale genotyping applications in foxtail millet [Setaria italica (L.)]. DNA Research. 2013;20(2):197–207. 10.1093/dnares/dst002. WOS:000318584600008. 23382459 PMC3628449

[36] Kumari K, Muthamilarasan M, Misra G, Gupta S, Subramanian A, Parida SK, et al Development of eSSR-markers in Setaria italica and their applicability in studying genetic diversity, cross-transferability and comparative mapping in millet and non-millet species. Plos One. 2013;8(6). UNSP e67742 10.1371/journal.pone.0067742. WOS:000320846500153. 23805325 PMC3689721

[37] Fukunaga K, Wang ZM, Kato K, Kawase M. Geographical variation of nuclear genome RFLPs and genetic differentiation in foxtail millet, Setaria italica (L.) P. Beauv. Genetic Resources and Crop Evolution. 2002;49(1):95–101. ISI:000173344600012.

[38] Fukunaga K, Domon E, Kawase M. Ribosomal DNA variation in foxtail millet, Setaria italica (L.) P. Beauv., and a survey of variation from Europe and Asia. Theoretical and Applied Genetics. 1997;95(5–6):751–6. ISI:A1997YF71500004.

[39] Schontz D, Rether B. Genetic variability in foxtail millet, Setaria italica (L.) P. Beauv.—RFLP using a heterologous rDNA probe. Pl Breed. 1998;117(3):231–4. ISI:000075128600006.

[40] Benabdelmouna A, Abirached-Darmency M, Darmency H. Phylogenetic and genomic relationships in *Setaria italica* and its close relatives based on the molecular diversity and chromosomal organization of 5S and 18S-5.8S-25S rDNA genes. Theoretical and Applied Genetics. 2001;103(5):668–77. ISI:000171839800002.

[41] Doyle JJ, Doyle JL. A rapid DNA isolation procedure for small quantities of fresh leaf material. Phytochemical Bulletin. 1987;19:11–5. Epub 15.

[42] Erland S, Henrion B, Martin F, Glover LA, Alexander IJ. Identification of the ectomycorrhizal basidiomycete *Tylospora fibrillosa* Donk by RFLP analysis of the PCR-amplified ITS and IGS regions of ribosomal DNA. New Phytol. 1994;126(3):525–32. ISI:A1994NG79800010. 10.1111/j.1469-8137.1994.tb04251.x 33874472

[43] Thompson JD, Gibson TJ, Plewniak F, Jeanmougin F, Higgins DG. The CLUSTAL_X windows interface: flexible strategies for multiple sequence alignment aided by quality analysis tools. Nucleic Acids Res. 1997;25(24):4876–82. ISI:000071498400004. 9396791 10.1093/nar/25.24.4876 PMC147148

[44] Toleno DM, Morrell PL, Clegg MT. Error detection in SNP data by considering the likelihood of recombinational history implied by three-site combinations. Bioinformatics. 2007;23(14):1807–14. 10.1093/bioinformatics/btm260. ISI:000249248300013. 17510172

[45] Thornton K. libsequence: a C++ class library for evolutionary genetic analysis. Bioinformatics. 2003;19(17):2325–7. 10.1093/bioinformatics/btg316. ISI:000186919200024. 14630667

[46] Watterson GA. Number of segregating sites in genetic models without recombination. Theoretical Population Biology. 1975;7(2):256–76. ISI:A1975W295600011. 1145509 10.1016/0040-5809(75)90020-9

[47] Tajima F. Evolutionary relationship of DNA sequences in finite populations. Genetics. 1983;105(2):437–60. ISI:A1983RH58800014. 6628982 10.1093/genetics/105.2.437 PMC1202167

[48] Tajima F. Statistical method for testing the neutral mutation hypothesis by DNA polymorphism. Genetics. 1989;123(3):585–95. 2513255 10.1093/genetics/123.3.585 PMC1203831

[49] Hudson R, Boos DD, Kaplan N. A statistical test for detecting geographic subdivision. Molecular biology and evolution. 1992;9(1):138–51. 1552836 10.1093/oxfordjournals.molbev.a040703

[50] Hudson RR. A new statistic for detecting genetic differentiation. Genetics. 2000;155(4):2011–4. ISI:000088664500043. 10924493 10.1093/genetics/155.4.2011 PMC1461195

[51] Wachowiak W, Balk PA, Savolainen O. Search for nucleotide diversity patterns of local adaptation in dehydrins and other cold-related candidate genes in Scots pine (Pinus sylvestris L.). Tree Genetics & Genomes. 2008;5(1):117–32. 10.1007/s11295-008-0188-3

[52] Pritchard JK, Stephens M, Donnelly P. Inference of population structure using multilocus genotype data. Genetics. 2000;155(2):945–59. ISI:000087475100039. 10835412 10.1093/genetics/155.2.945 PMC1461096

[53] Falush D, Stephens M, Pritchard JK. Inference of population structure using multilocus genotype data: Linked loci and correlated allele frequencies. Genetics. 2003;164(4):1567–87. ISI:000185248000029. 12930761 10.1093/genetics/164.4.1567 PMC1462648

[54] Evanno G, Regnaut S, Goudet J. Detecting the number of clusters of individuals using the software STRUCTURE: a simulation study. Molecular Ecology. 2005;14(8):2611–20. 10.1111/j.1365-294X.2005.02553.x. ISI:000229961500029. 15969739

[55] Earl DA. STRUCTURE HARVESTER: a website and program for visualizing STRUCTURE output and implementing the Evanno method. Conservation genetics resources. 2012;4(2):359–61.

[56] Andolfatto P, Przeworski M. A genome-wide departure from the standard neutral model in natural populations of *Drosophila* . Genetics. 2000;156(1):257–68. ISI:000089209800021. 10978290 10.1093/genetics/156.1.257 PMC1461228

[57] Hudson RR. Estimating the recombination parameter of a finite population-model without selection. Genetical Research. 1987;50(3):245–50. ISI:A1987L886800012. 3443297 10.1017/s0016672300023776

[58] Hudson RR. Two-locus sampling distributions and their application. Genetics. 2001;159(4):1805–17. ISI:000173106800035. 11779816 10.1093/genetics/159.4.1805 PMC1461925

[59] Wall JD. Recombination and the power of statistical tests of neutrality. Genetical Research. 1999;74(1):65–79. ISI:000082450800007.

[60] Hudson RR, Kaplan NL. Statistical properties of the number of recombination events in the history of a sample of DNA sequences. Genetics. 1985;111(1):147–64. ISI:A1985APL1600011. 4029609 10.1093/genetics/111.1.147 PMC1202594

[61] Weir BS, Cockerham CC. Estimating F-statistics for the analysis of population structure. Evolution. 1984;38(6):1358–70. ISI:A1984TY40400017. 28563791 10.1111/j.1558-5646.1984.tb05657.x

[62] Remington DL, Thornsberry JM, Matsuoka Y, Wilson LM, Whitt SR, Doeblay J, et al Structure of linkage disequilibrium and phenotypic associations in the maize genome. Proceedings of the National Academy of Sciences of the United States of America. 2001;98(20):11479–84. ISI:000171237100090. 11562485 10.1073/pnas.201394398 PMC58755

[63] Li PH, Brutnell TP. *Setaria viridis* and *Setaria italica*, model genetic systems for the Panicoid grasses. J Exp Bot. 2011;62(9):3031–7. 10.1093/Jxb/Err096. ISI:000291526500005. 21459768

[64] Rakshit S, Rakshit A, Matsumura H, Takahashi Y, Hasegawa Y, Ito A, et al Large-scale DNA polymorphism study of *Oryza sativa* and O-rufipogon reveals the origin and divergence of Asian rice. Theoretical and Applied Genetics. 2007;114(4):731–43. 10.1007/s00122-006-0473-1. ISI:000243973300015. 17219210

[65] Liu ZL, Bai GH, Zhang DD, Zhu CS, Xia XY, Cheng RH, et al Genetic diversity and population structure of elite foxtail millet [Setaria italica (L.) P. Beauv.] germplasm in China. Crop Science. 2011;51(4):1655–63. 10.2135/cropsci2010.11.0643. ISI:000291972900028.

[66] Liu ZL, Zhang T, Li C, Bai GH. Genetic diversity and classification of cytoplasm of Chinese elite foxtail millet [Setaria italica (L.) P. Beauv.] germplasm. Crop Science. 2014;54(2):659–66. 10.2135/cropsci2012.11.0646. WOS:000336746800023.

[67] Kihara H, Kishimoto E. Bastarde zwischen *Setaria italica* und *S*. *viridis* . Botanical Magazine. 1942;56:62–7.

[68] Li HW, Li CH, Pao WK. Cytological and genetical studies of the interspecific cross of the cultivated foxtail millet, Setaria italica (L.) Beauv., and the green foxtail millet, S. viridis L. Journal of the American Society of Agronomy. 1945;31(1):32–54.

[69] Wang R-l, Wendel JF, Dekker JH. Weedy adaptation in *Setaria* spp. I. Isozyme analysis of genetic diversity and population genetic structure in *Setaria viridis* . American Journal of Botany. 1995:308–17.

[70] Lu HY, Zhang JP, Liu KB, Wu NQ, Li YM, Zhou KS, et al Earliest domestication of common millet (Panicum miliaceum) in East Asia extended to 10,000 years ago. Proceedings of the National Academy of Sciences of the United States of America. 2009;106(18):7367–72. 10.1073/pnas.0900158106. ISI:000265783600021. 19383791 PMC2678631

[71] Turrill WB. Studies on the origin of cultivated plants. Nature. 1926;118:392–3. ISI:000188314100197.

[72] Kawase M, Sakamoto S. Geographical distribution and fenetic analysis of phenol color-reaction in foxtail millet, Setaria italica (L.) P. Beauv. Theoretical and Applied Genetics. 1982;63(2):117–9. ISI:A1982PL25300004. 10.1007/BF00303690 24270757

[73] Nordborg M, Hu TT, Ishino Y, Jhaveri J, Toomajian C, Zheng HG, et al The pattern of polymorphism in *Arabidopsis thaliana* . Plos Biol. 2005;3(7):1289–99. ARTN e196 10.1371/journal.pbio.0030196. ISI:000230759000016. PMC1135296 15907155

[74] Kim S, Plagnol V, Hu TT, Toomajian C, Clark RM, Ossowski S, et al Recombination and linkage disequilibrium in *Arabidopsis thaliana* . Nature Genetics. 2007;39(9):1151–5. 10.1038/Ng2115. ISI:000249122400027. 17676040

[75] Mather KA, Caicedo AL, Polato NR, Olsen KM, McCouch S, Purugganan MD. The extent of linkage disequilibrium in rice (Oryza sativa L.). Genetics. 2007;177(4):2223–32. 10.1534/genetics.107.079616. ISI:000251949800022. 17947413 PMC2219496

[76] Morrell PL, Clegg MT. Genetic evidence for a second domestication of barley (Hordeum vulgare) east of the Fertile Crescent. Proceedings of the National Academy of Sciences of the United States of America. 2007;104(9):3289–94. 10.1073/pnas.0611377104. ISI:000244661400049. 17360640 PMC1805597

[77] Morrell PL, Toleno DM, Lundy KE, Clegg MT. Low levels of linkage disequilibrium in wild barley (Hordeum vulgare ssp spontaneum) despite high rates of self-fertilization. P Natl Acad Sci USA. 2005;102(7):2442–7. 10.1073/pnas.0409804102. ISI:000227073100036. PMC549024 15699350

[78] Lin JZ, Morrell PL, Clegg MT. The influence of linkage and inbreeding on patterns of nucleotide sequence diversity at duplicate alcohol dehydrogenase loci in wild barley (Hordeum vulgare ssp spontaneum). Genetics. 2002;162(4):2007–15. ISI:000180502300041. 12524366 10.1093/genetics/162.4.2007 PMC1462393

[79] Bakker EG, Stahl EA, Toomajian C, Nordborg M, Kreitman M, Bergelson J. Distribution of genetic variation within and among local populations of *Arabidopsis thaliana* over its species range. Molecular Ecology. 2006;15(5):1405–18. 10.1111/j.1365-294X.2006.02884.x. ISI:000236584900016. 16626462

[80] Keightley PD, Otto SP. Interference among deleterious mutations favours sex and recombination in finite populations. Nature. 2006;443(7107):89–92. 10.1038/Nature05049. ISI:000240313900045. 16957730

[81] Martin G, Otto SP, Lenormand T. Selection for recombination in structured populations. Genetics. 2006;172(1):593–609. 10.1534/genetics.104.039982. ISI:000235197700052. 15944358 PMC1456186

[82] Vetriventhan M, Upadhyaya HD, Anandakumar CR, Senthilvel S, Varshney RK, Parzies HK. Population structure and linkage disequilibrium of ICRISAT foxtail millet (Setaria italica (L.) P. Beauv.) core collection. Euphytica. 2014;196(3):423–35. 10.1007/s10681-013-1044-6. WOS:000332603800010.

[83] Wang CF, Jia GQ, Zhi H, Niu ZG, Chai Y, Li W, et al Genetic diversity and population structure of Chinese foxtail millet [Setaria italica (L.) Beauv.] landraces. G3-Genes Genom Genet. 2012;2(7):769–77. 10.1534/g3.112.002907. WOS:000312454800006. PMC3385983 22870400

