# Supplementary figures and images for: Nucleotide Sequence Diversity and Linkage Disequilibrium of Four Nuclear Loci in Foxtail Millet (Setaria italica)
Source: PLoS One. 2015 Sep 1;10(9):e0137088. doi: 10.1371/journal.pone.0137088 (PMC4556640; doi:10.1371/journal.pone.0137088)

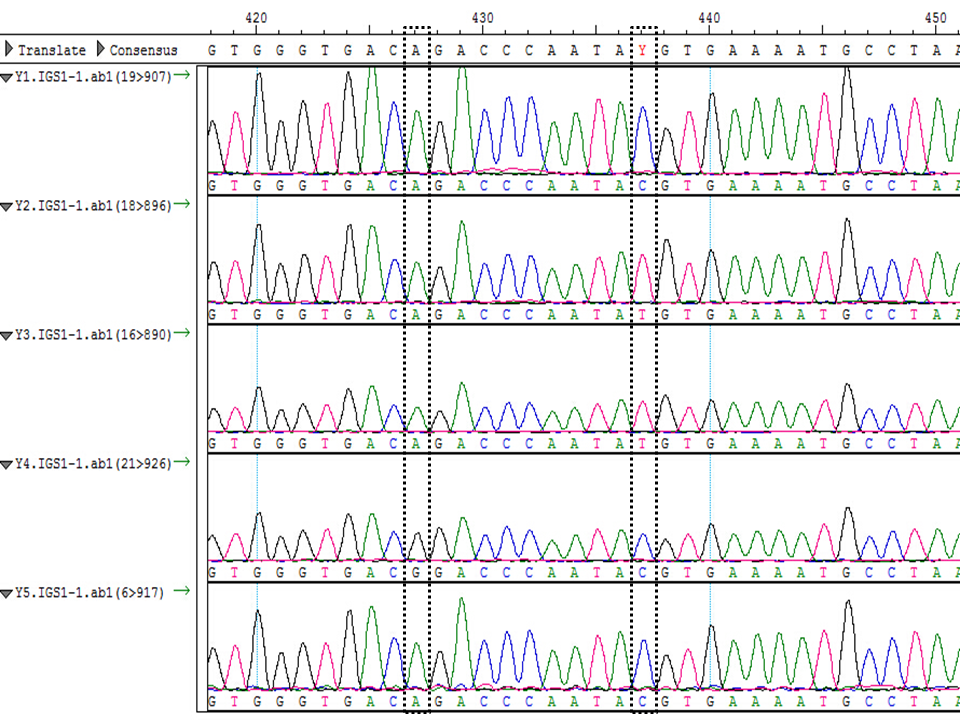

Supplement: S1 Fig — (TIF) [file pone.0137088.s001.tif]
