# Supplementary material for: Nucleotide Sequence Diversity and Linkage Disequilibrium of Four Nuclear Loci in Foxtail Millet (Setaria italica)
Source: PLoS One. 2015 Sep 1;10(9):e0137088. doi: 10.1371/journal.pone.0137088 (PMC4556640; doi:10.1371/journal.pone.0137088)
Supplement: S1 Table — GG = geographic group. (PDF) [file pone.0137088.s002.pdf]

Table S1 Sampling and haplotype information for domesticated foxtail millet.

Accession no	Accession no2	Locality	latitude	Longitude	GG
Y005	I7T5	Afghanistan, Kabul	34.515500 N	69.195194 E	CA
Y008	I10T8	Afghanistan	33.939111 N	67.709953 E	CA
Y028	I35T28	Afghanistan	33.939111 N	67.709953 E	CA
Y030	I38T30	Afghanistan	33.939111 N	67.709953 E	CA
Y034	I42T34	Afghanistan	33.939111 N	67.709953 E	CA
Y035	I43T35	Afghanistan	33.939111 N	67.709953 E	CA
Y037	I46T37	Afghanistan	33.939111 N	67.709953 E	CA
Y039	I51T39	Afghanistan	33.939111 N	67.709953 E	CA
Y040	I52T40	Afghanistan	33.939111 N	67.709953 E	CA
Y042	I54T42	Afghanistan	33.939111 N	67.709953 E	CA
Y048	I60T48	Afghanistan	33.939111 N	67.709953 E	CA
Y049	I61T49	Afghanistan	33.939111 N	67.709953 E	CA
Y050	I62T50	Afghanistan	33.939111 N	67.709953 E	CA
Y052	I64T52	Afghanistan	33.939111 N	67.709953 E	CA
Y053	I65T53	Afghanistan	33.939111 N	67.709953 E	CA
Y195	J222990	Afghanistan	33.939111 N	67.709953 E	CA
Y211	J225323	Afghanistan	33.939111 N	67.709953 E	CA
Y196	J222995	Kyrgyzstan	41.204381 N	74.766097 E	CA
Y213	J225325	Kyrgyzstan	41.204381 N	74.766097 E	CA
Y002	I4T2	Kazakhstan	48.019572 N	66.923683 E	CA
Y218	J225330	Kazakhstan	48.019572 N	66.923683 E	CA
Y219	J225331	Kazakhstan	48.019572 N	66.923683 E	CA
Y220	J225332	Kazakhstan	48.019572 N	66.923683 E	CA
Y221	J225333	Kazakhstan	48.019572 N	66.923683 E	CA
Y197	J222996	Uzbekistan	41.377492 N	64.585261 E	CA
Y177	J222583	Bangladesh	23.684994 N	90.356331 E	CA
Y178	J222584	Bangladesh	23.684994 N	90.356331 E	CA
Y179	J222585	Bangladesh	23.684994 N	90.356331 E	CA
Y079	I91T79	Nepal	23.684994 N	90.356331 E	CA
Y080	I92T80	Nepal	23.684994 N	90.356331 E	CA
Y152	J36270	Nepal	23.684994 N	90.356331 E	CA
Y153	J36280	Nepal	23.684994 N	90.356331 E	CA
Y154	J36288	Nepal	23.684994 N	90.356331 E	CA
Y194	J222969	Nepal	23.684994 N	90.356331 E	CA
Y020	I22T20	India	20.593683 N	78.962881 E	SA
Y021	I23T21	India	20.593683 N	78.962881 E	SA
Y026	I28T26	India	20.593683 N	78.962881 E	SA

Y047	I59T47	India	20.593683 N	78.962881 E	SA
Y061	I73T61	India, Himachal Pradesh	20.593683 N	78.962881 E	SA
Y062	I74T62	India	20.593683 N	78.962881 E	SA
Y077	I89T77	India	20.593683 N	78.962881 E	SA
Y078	I90T78	India	20.593683 N	78.962881 E	SA
Y096	I109T96	India, Andhra, Pradesh	17.047761 N	80.098186 E	SA
Y102	I115T102	India	20.593683 N	78.962881 E	SA
Y112	I125T112	India	20.593683 N	78.962881 E	SA
Y166	J105631	India	20.593683 N	78.962881 E	SA
Y167	J105634	India	20.593683 N	78.962881 E	SA
Y168	J105636	India	20.593683 N	78.962881 E	SA
Y169	J105637	India	17.047761 N	80.098186 E	SA
s10	14745	India	20.593683 N	78.962881 E	SA
s11	14769	India	20.593683 N	78.962881 E	SA
s12	14770	India	20.593683 N	78.962881 E	SA
s9	14744	India	20.593683 N	78.962881 E	SA
Y031	I39T31	Pakistan	30.375322 N	69.345117 E	CA
Y032	I40T32	Pakistan	30.375322 N	69.345117 E	CA
Y045	I57T45	Pakistan	30.375322 N	69.345117 E	CA
Y055	I67T55	Pakistan	30.375322 N	69.345117 E	CA
Y057	I69T57	Pakistan	30.375322 N	69.345117 E	CA
Y058	I70T58	Pakistan	30.375322 N	69.345117 E	CA
Y059	I71T59	Pakistan	30.375322 N	69.345117 E	CA
Y060	I72T60	Pakistan	30.375322 N	69.345117 E	CA
Y156	J71550	Pakistan	30.375322 N	69.345117 E	CA
Y157	J71580	Pakistan	30.375322 N	69.345117 E	CA
Y160	J73884	Pakistan	30.375322 N	69.345117 E	CA
Y161	J73893	Pakistan	30.375322 N	69.345117 E	CA
Y162	J73903	Pakistan	30.375322 N	69.345117 E	CA
Y041	I53T41	Iran	32.427908 N	53.688047 E	NE
Y006	I8T6	Iran	32.427908 N	53.688047 E	NE
Y007	I9T7	Iran	32.427908 N	53.688047 E	NE
Y036	I45T36	Iran	32.427908 N	53.688047 E	NE
Y038	I50T38	Iran	32.427908 N	53.688047 E	NE
Y044	I56T44	Iran	32.427908 N	53.688047 E	NE
Y046	I58T46	Iran	32.427908 N	53.688047 E	NE
Y081	I93T81	Iran	32.427908 N	53.688047 E	NE
Y097	I110T97	Lebanon	32.427908 N	53.688047 E	NE
Y098	I111T98	Lebanon	32.427908 N	53.688047 E	NE
Y099	I112T99	Lebanon	32.427908 N	53.688047 E	NE
Y100	I113T100	Lebanon	32.427908 N	53.688047 E	NE

Y113	I126T113	Lebanon	32.427908 N	53.688047 E	NE
Y114	I127T114	Lebanon	32.427908 N	53.688047 E	NE
Y115	I128T115	Lebanon	32.427908 N	53.688047 E	NE
Y116	I129T116	Lebanon	32.427908 N	53.688047 E	NE
Y117	I130T117	Lebanon	32.427908 N	53.688047 E	NE
Y118	I131T118	Lebanon	32.427908 N	53.688047 E	NE
Y119	I132T119	Lebanon	32.427908 N	53.688047 E	NE
Y120	I133T120	Lebanon	32.427908 N	53.688047 E	NE
s40	14960	Lebanon	32.427908 N	53.688047 E	NE
s41	14961	Lebanon	32.427908 N	53.688047 E	NE
s42	14962	Lebanon	32.427908 N	53.688047 E	NE
s43	14963	Lebanon	32.427908 N	53.688047 E	NE
Y013	I15T13	Turkey	38.963744 N	35.243322 E	NE
Y014	I16T14	Turkey	38.963744 N	35.243322 E	NE
Y015	I17T15	Turkey	38.963744 N	35.243322 E	NE
Y017	I19T17	Turkey	38.963744 N	35.243322 E	NE
Y018	I20T18	Turkey	38.963744 N	35.243322 E	NE
Y019	I21T19	Turkey	38.963744 N	35.243322 E	NE
Y022	I24T22	Turkey	38.963744 N	35.243322 E	NE
Y023	I25T23	Turkey	38.963744 N	35.243322 E	NE
Y024	I26T24	Turkey	38.963744 N	35.243322 E	NE
Y025	I27T25	Turkey, Ankara	39.943872 N	32.856033 E	NE
Y027	I34T27	Turkey	38.963744 N	35.243322 E	NE
Y051	I63T51	Turkey, Ankara	39.943872 N	32.856033 E	NE
Y150	J25198	Kagaiva	36.204825 N	138.252925 E	KJ
Y151	J36265	Iwate	39.703619 N	141.319350 E	KJ
Y155	J69743	Miyazaki	31.907675 N	131.420242 E	KJ
Y158	J71623	Gunma	36.390667 N	139.060406 E	KJ
Y159	J71633	Nara	34.685083 N	135.805000 E	KJ
Y164	J84035	Ussr	39.703619 N	141.319350 E	KJ
Y165	J84060	Hokkaido	31.907675 N	131.420242 E	KJ
Y170	J108625	Shizuoka	36.390667 N	139.060406 E	KJ
Y190	J222829	Hyougo	34.691269 N	135.183072 E	KJ
s1	14691	Japan	36.204825 N	138.252925 E	KJ
s2	14692	Japan	36.204825 N	138.252925 E	KJ
s3	14693	Japan	36.204825 N	138.252925 E	KJ
s4	14695	Japan	36.204825 N	138.252925 E	KJ
Y173	J222568	PEP, Korea	35.907756 N	127.766922 E	KJ
Y185	J222702	PEP, Korea	35.907756 N	127.766922 E	KJ
Y186	J222711	PEP, Korea	35.907756 N	127.766922 E	KJ
Y187	J222734	PEP, Korea	35.907756 N	127.766922 E	KJ

Y188	J222741	PEP, Korea	35.907756 N	127.766922 E	KJ
Y189	J222752	PEP, Korea	35.907756 N	127.766922 E	KJ
s5	14739	North Korea	35.907756 N	127.766922 E	KJ
s6	14740	North Korea	35.907756 N	127.766922 E	KJ
s7	14742	North Korea	35.907756 N	127.766922 E	KJ
s8	14743	North Korea	35.907756 N	127.766922 E	KJ
Y082	I94T82	Taiwan	23.697811 N	120.960514 E	Ch
Y083	I95T83	Taiwan	23.697811 N	120.960514 E	Ch
Y084	I96T84	Taiwan	23.697811 N	120.960514 E	Ch
Y085	I97T85	Taiwan	23.697811 N	120.960514 E	Ch
Y086	I98T86	Taiwan	23.697811 N	120.960514 E	Ch
Y087	I99T87	Taiwan	23.697811 N	120.960514 E	Ch
Y088	I100T88	Taiwan	23.697811 N	120.960514 E	Ch
Y089	I101T89	Taiwan	23.697811 N	120.960514 E	Ch
Y090	I102T90	Taiwan	23.697811 N	120.960514 E	Ch
Y091	I103T91	Taiwan	23.697811 N	120.960514 E	Ch
Y092	I104T92	Taiwan	23.697811 N	120.960514 E	Ch
Y093	I105T93	Taiwan	23.697811 N	120.960514 E	Ch
Y094	I106T94	Taiwan	23.697811 N	120.960514 E	Ch
Y163	J73912	Taiwan	23.697811 N	120.960514 E	Ch
Y172	J222567	Taiwan	23.697811 N	120.960514 E	Ch
Y180	J222589	Taiwan	23.697811 N	120.960514 E	Ch
s62	14595	Shanxi,China	34.261683 N	108.949028 E	Ch
s63	14590	Shanxi,China	34.261683 N	108.949028 E	Ch
s64	14588	Shanxi,China	34.261683 N	108.949028 E	Ch
s66	14575	Shanxi,China	34.261683 N	108.949028 E	Ch
s67	44	Heilongjiang,China	45.741492 N	126.643342 E	Ch
s70	41	Heilongjiang,China	45.741492 N	126.643342 E	Ch
s82	42	Heilongjiang,China	45.741492 N	126.643342 E	Ch
s83	43	Heilongjiang,China	45.741492 N	126.643342 E	Ch
s59	5811	Shanxi(east),China	37.871114 N	112.569350 E	Ch
s60	5814	Shanxi(east),China	37.871114 N	112.569350 E	Ch
s84	5812	Shanxi(east),China	37.871114 N	112.569350 E	Ch
s87	5813	Shanxi(east),China	37.871114 N	112.569350 E	Ch
s61	1300	Liaoning,China	41.796617 N	123.411683 E	Ch
s85	1298	Liaoning,China	41.796617 N	123.411683 E	Ch
s86	1299	Liaoning,China	41.796617 N	123.411683 E	Ch
s88	1301	Liaoning,China	41.796617 N	123.411683 E	Ch
s68	11051	Shandong,China	36.667072 N	117.005600 E	Ch
s69	11053	Shandong,China	36.667072 N	117.005600 E	Ch
s71	11052	Shandong,China	36.667072 N	117.005600 E	Ch

s72	11054	Shandong,China	36.667072 N	117.005600 E	Ch
s73	735	Jilin,China	43.873542 N	126.468794 E	Ch
s74	734	Jilin,China	43.873542 N	126.468794 E	Ch
s75	733	Jilin,China	43.873542 N	126.468794 E	Ch
s76	731	Jilin,China	43.873542 N	126.468794 E	Ch
s65	18542	Gansu,China	36.068039 N	103.750053 E	Ch
s79	18571	Gansu,China	36.068039 N	103.750053 E	Ch
s80	18541	Gansu,China	36.068039 N	103.750053 E	Ch
s81	18569	Gansu,China	36.068039 N	103.750053 E	Ch
s58	12102	Hebei,China	38.045128 N	114.489778 E	Ch
s77	12104	Hebei,China	38.045128 N	114.489778 E	Ch
s78	12103	Hebei,China	38.045128 N	114.489778 E	Ch
s32	14614	Jiangsu,China	32.047614 N	118.772781 E	Ch
s52	15070	Jiangsu,China	32.047614 N	118.772781 E	Ch
s53	15079	Jiangsu,China	32.047614 N	118.772781 E	Ch
s54	15086	Jiangsu,China	32.047614 N	118.772781 E	Ch
s56	1604	Neimonggu,China	40.820942 N	111.663300 E	Ch
s57	1603	Neimonggu,China	40.820942 N	111.663300 E	Ch
s90	1605	Neimonggu,China	40.820942 N	111.663300 E	Ch
s91	1602	Neimonggu,China	40.820942 N	111.663300 E	Ch
s24	14606	Xinjiang,China	43.790939 N	873.106117 E	Ch
s25	14607	Xinjiang,China	43.790939 N	873.106117 E	Ch
s26	14608	Xinjiang,China	43.790939 N	873.106117 E	Ch
s27	14609	Xinjiang,China	43.790939 N	873.106117 E	Ch
s33	14615	Anhui,China	31.863256 N	117.275703 E	Ch
s34	14616	Zhejiang,China	30.265994 N	120.159247 E	Ch
s35	14617	Zhejiang,China	30.265994 N	120.159247 E	Ch
s116	14618	Fujian,China	26.078589 N	119.297814 E	Ch
s36	14620	Hunan,China	28.200825 N	112.981269 E	Ch
s37	14623	Hunan,China	28.200825 N	112.981269 E	Ch
s38	14624	Hunan,China	28.200825 N	112.981269 E	Ch
s39	14625	Hunan,China	28.200825 N	112.981269 E	Ch
s117	14627	Hainan,China	20.031794 N	110.346511 E	Ch
s118	14628	Hainan,China	20.031794 N	110.346511 E	Ch
s119	14629	Hainan,China	20.031794 N	110.346511 E	Ch
s120	14630	Hainan,China	20.031794 N	110.346511 E	Ch
s108	14640	Guangxi,China	22.806542 N	108.311769 E	Ch
s109	14650	Guangxi,China	22.806542 N	108.311769 E	Ch
s110	14652	Guangxi,China	22.806542 N	108.311769 E	Ch
s111	14653	Guangxi,China	22.806542 N	108.311769 E	Ch
s112	14661	Yunnan,China	25.043844 N	102.704567 E	Ch

s113	14662	Yunnan,China	25.043844 N	102.704567 E	Ch
s114	14663	Yunnan,China	25.043844 N	102.704567 E	Ch
s115	14668	Yunnan,China	25.043844 N	102.704567 E	Ch
s18	18740	Nixia,China	38.468011 N	106.271942 E	Ch
s19	18747	Nixia,China	38.468011 N	106.271942 E	Ch
s20	18751	Nixia,China	38.468011 N	106.271942 E	Ch
s13	14671	Tibet,China	29.657589 N	91.132050 E	Ch
s14	14672	Tibet,China	29.657589 N	91.132050 E	Ch
s15	14673	Tibet,China	29.657589 N	91.132050 E	Ch
s16	14674	Tibet,China	29.657589 N	91.132050 E	Ch
s28	22309	Tibet,China	29.657589 N	91.132050 E	Ch
s29	22312	Tibet,China	29.657589 N	91.132050 E	Ch
s30	22313	Tibet,China	29.657589 N	91.132050 E	Ch
s31	22315	Tibet,China	29.657589 N	91.132050 E	Ch
s21	25655	Qinhai,China	36.609447 N	101.787453 E	Ch
s22	25658	Qinhai,China	36.609447 N	101.787453 E	Ch
s23	25684	Qinhai,China	36.609447 N	101.787453 E	Ch
s55	18759	Qinhai,China	36.609447 N	101.787453 E	Ch
s104	25867	Guizhou,China	26.576875 N	106.711372 E	Ch
s105	25868	Guizhou,China	26.576875 N	106.711372 E	Ch
s106	25869	Guizhou,China	26.576875 N	106.711372 E	Ch
s107	25885	Guizhou,China	26.576875 N	106.711372 E	Ch
s89	18875	Zhangjikou Hubei,China	40.567219 N	115.331983 E	Ch
Y208	J223009	Mongolia	46.862497 N	103.846656 E	Ch
Y174	J222569	Philippines	12.879722 N	121.774017 E	SEA
Y182	J222620	Philippines	12.879722 N	121.774017 E	SEA
Y183	J222624	Philippines	12.879722 N	121.774017 E	SEA
Y184	J222626	Philippines	12.879722 N	121.774017 E	SEA
Y191	J222963	Philippines	12.879722 N	121.774017 E	SEA
Y192	J222965	Philippines	12.879722 N	121.774017 E	SEA
Y193	J222966	Philippines	12.879722 N	121.774017 E	SEA
Y175	J222580	Indonesia	0.789280 S	113.921328 E	SEA
Y176	J222582	Indonesia	0.789280 S	113.921328 E	SEA
Y001	I1T1	U.S.A., Missouri	37.964253 N	91.831833 W	NA
Y010	I12T10	U.S.A., Texas	31.968600 N	99.901814 W	NA
Y011	I13T11	U.S.A., South Dakota	37.090239 N	95.712892 W	NA
Y012	I14T12	U.S.A., Nebraska	41.492536 N	99.901814 W	NA
Y072	I84T72	Canada, Ontario	37.964253 N	91.831833 W	NA
Y076	I88T76	U.S.A., Colorado	37.090239 N	95.712892 W	NA
Y210	J223012	Georgia	32.157436 N	82.907122 W	NA
Y225	J225337	Yugoslavia	32.157436 N	82.907122 W	NA

Y209	J223010	Bulgaria	42.733883 N	25.485831 E	EU
Y063	I75T63	Spain	40.463667 N	3.749219 W	EU
Y199	J222998	Spain	40.463667 N	3.749219 W	EU
s48	14981	Romania	45.943161 N	24.966761 E	EU
s49	14982	Romania	45.943161 N	24.966761 E	EU
s50	14983	Romania	45.943161 N	24.966761 E	EU
s51	14984	Romania	45.943161 N	24.966761 E	EU
Y064	I76T64	Hungary	47.162494 N	19.503303 E	EU
Y065	I77T65	Hungary	47.162494 N	19.503303 E	EU
Y066	I78T66	Hungary	47.162494 N	19.503303 E	EU
Y067	I79T67	Hungary	47.162494 N	19.503303 E	EU
Y068	I80T68	Hungary	47.162494 N	19.503303 E	EU
Y069	I81T69	Hungary	47.162494 N	19.503303 E	EU
Y070	I82T70	Hungary	47.162494 N	19.503303 E	EU
Y071	I83T71	Hungary	47.162494 N	19.503303 E	EU
Y121	I134T121	Hungary	47.162494 N	19.503303 E	EU
Y124	I137T124	Hungary	47.162494 N	19.503303 E	EU
Y125	I138T125	Hungary	47.162494 N	19.503303 E	EU
Y126	I139T126	Hungary	47.162494 N	19.503303 E	EU
Y224	J225336	Hungary	47.162494 N	19.503303 E	EU
s92	14971	Hungary	47.162494 N	19.503303 E	EU
s93	14972	Hungary	47.162494 N	19.503303 E	EU
s94	14973	Hungary	47.162494 N	19.503303 E	EU
s95	14974	Hungary	47.162494 N	19.503303 E	EU
Y003	I5T3	Germany, Saxony	51.165692 N	10.451525 E	EU
Y200	J222999	Germany	51.165692 N	10.451525 E	EU
s44	15029	Germany	51.165692 N	10.451525 E	EU
s45	15030	Germany	51.165692 N	10.451525 E	EU
s46	15031	Germany	51.165692 N	10.451525 E	EU
s47	15032	Germany	51.165692 N	10.451525 E	EU
Y073	I85T73	Former, Soviet, Union	61.524011 N	105.318756 E	EU
Y074	I86T74	Former, Soviet, Union	61.524011 N	105.318756 E	EU
Y075	I87T75	Former, Soviet, Union	61.524011 N	105.318756 E	EU
Y101	I114T101	Former, Soviet, Union	61.524011 N	105.318756 E	EU
Y123	I136T123	Poland	51.919439 N	19.145136 E	EU
Y111	I124T111	Switzerland	46.818189 N	8.227511 E	EU
Y095	I107T95	Belgium	50.503886 N	4.469936 E	EU
Y201	J223000	Belgium	50.503886 N	4.469936 E	EU
Y004	I6T4	France	46.227639 N	2.213750 E	EU
Y203	J223002	France	46.227639 N	2.213750 E	EU
Y204	J223003	France	46.227639 N	2.213750 E	EU

Y205	J223004	France	46.227639 N	2.213750 E	EU
s100	14995	France	46.227639 N	2.213750 E	EU
s101	14996	France	46.227639 N	2.213750 E	EU
s102	14997	France	46.227639 N	2.213750 E	EU
s103	14998	France	46.227639 N	2.213750 E	EU
s96	14986	Holland	52.132633 N	5.291267 E	EU
s97	14987	Holland	52.132633 N	5.291267 E	EU
s98	14988	Holland	52.132633 N	5.291267 E	EU
Y206	J223007	Ukraine	48.379433 N	31.165581 E	EU
Y214	J225326	Ukraine	48.379433 N	31.165581 E	EU
Y215	J225327	Ukraine	48.379433 N	31.165581 E	EU
Y216	J225328	Ukraine	48.379433 N	31.165581 E	EU
Y217	J225329	Ukraine	48.379433 N	31.165581 E	EU
Y198	J222997	Russia	61.524011 N	105.318756 E	EU
Y207	J223008	Russia	61.524011 N	105.318756 E	EU
Y212	J225324	Russia	61.524011 N	105.318756 E	EU
Y222	J225334	Russia	61.524011 N	105.318756 E	EU
Y223	J225335	Russia	61.524011 N	105.318756 E	EU
Y103	I116T103	Ethiopia	9.145000 S	40.489672 E	EAF
Y043	I55T43	Kenya	0.023558 S	37.906194 E	EAF
Y107	I120T107	Kenya	0.023558 S	37.906194 E	EAF
Y108	I121T108	Kenya	0.023558 S	37.906194 E	EAF
Y109	I122T109	Kenya	0.023558 S	37.906194 E	EAF
Y110	I123T110	Kenya	0.023558 S	37.906194 E	EAF
Y171	J222566	Kenya	0.023558 S	37.906194 E	EAF
Y009	I11T9	Morocco, Ouarzazate	31.791703 S	7.092619 W	WAF
Y122	I135T122	Morocco	30.918889 S	6.903819 W	WAF
Y029	I36T29	South Africa, Transvaal	30.559483 S	22.937506 E	SAF
Y105	I118T105	South Africa	28.725000 S	24.769100 E	SAF

GG = geographic group.
