# Supplementary material for: Nucleotide Sequence Diversity and Linkage Disequilibrium of Four Nuclear Loci in Foxtail Millet (Setaria italica)
Source: PLoS One. 2015 Sep 1;10(9):e0137088. doi: 10.1371/journal.pone.0137088 (PMC4556640; doi:10.1371/journal.pone.0137088)
Supplement: S2 Table — (PDF) [file pone.0137088.s003.pdf]

Table S2. accession numbers of haplotypes.

gene	haplotype	accession numbers
G3PDH	Hap1	KT335428
G3PDH	Hap2	KT335429
G3PDH	Hap3	KT335430
G3PDH	Hap4	KT335431
G3PDH	Hap5	KT335432
G3PDH	Hap6	KT335433
G3PDH	Hap7	KT335434
G3PDH	Hap8	KT335435
G3PDH	Hap9	KT335436
G3PDH	Hap10	KT335437
G3PDH	Hap11	KT335438
G3PDH	Hap12	KT335439
G3PDH	Hap13	KT335440
G3PDH	Hap14	KT335441
G3PDH	Hap15	KT335442
G3PDH	Hap16	KT335443
G3PDH	Hap17	KT335444
G3PDH	Hap18	KT335445
G3PDH	Hap19	KT335446
G3PDH	Hap20	KT335447
G3PDH	Hap21	KT335448
G3PDH	Hap22	KT335449
IGS1	Hap1	KT335450
IGS1	Hap2	KT335451
IGS1	Hap3	KT335452
IGS1	Hap4	KT335453
IGS1	Hap5	KT335454
IGS1	Hap6	KT335455
IGS1	Hap7	KT335456
IGS1	Hap8	KT335457
IGS1	Hap9	KT335458
IGS1	Hap10	KT335459
IGS1	Hap11	KT335460
IGS1	Hap12	KT335461
IGS1	Hap13	KT335462
IGS1	Hap14	KT335463
IGS1	Hap15	KT335464
IGS1	Hap16	KT335465
IGS1	Hap17	KT335466
IGS1	Hap18	KT335467
IGS1	Hap19	KT335468
IGS1	Hap20	KT335469

IGS1	Hap21	KT335470
IGS1	Hap22	KT335471
IGS1	Hap23	KT335472
IGS1	Hap24	KT335473
IGS1	Hap25	KT335474
IGS1	Hap26	KT335475
IGS1	Hap27	KT335476
TPI1	Hap1	KT335477
TPI1	Hap2	KT335478
TPI1	Hap3	KT335479
TPI1	Hap4	KT335480
TPI1	Hap5	KT335481
TPI1	Hap6	KT335482
TPI1	Hap7	KT335483
TPI1	Hap8	KT335484
TPI1	Hap9	KT335485
TPI1	Hap10	KT335486
TPI1	Hap11	KT335487
TPI1	Hap12	KT335488
TPI1	Hap13	KT335489
TPI1	Hap14	KT335490
TPI1	Hap15	KT335491
TPI1	Hap16	KT335492
TPI1	Hap17	KT335493
TPI1	Hap18	KT335494
TPI1	Hap19	KT335495
TPI1	Hap20	KT335496
TPI1	Hap21	KT335497
TPI1	Hap22	KT335498
TPI1	Hap23	KT335499
TPI1	Hap24	KT335500
TPI1	Hap25	KT335501
TPI1	Hap26	KT335502
TPI1	Hap27	KT335503
TPI1	Hap28	KT335504
TPI1	Hap29	KT335505
TPI1	Hap30	KT335506
ADH1	Hap1	KT335507
ADH1	Hap2	KT335508
ADH1	Hap3	KT335509
ADH1	Hap4	KT335510
ADH1	Hap5	KT335511
ADH1	Hap6	KT335512
ADH1	Hap7	KT335513

ADH1	Hap8	KT335514
ADH1	Hap9	KT335515
ADH1	Hap10	KT335516
ADH1	Hap11	KT335517
ADH1	Hap12	KT335518
ADH1	Hap13	KT335519
ADH1	Hap14	KT335520
ADH1	Hap15	KT335521
ADH1	Hap16	KT335522
